# Combining a Density Gradient of Biomacromolecular Nanoparticles with Biological Effectors in an Electrospun Fiber‐Based Nerve Guidance Conduit to Promote Peripheral Nerve Repair

**DOI:** 10.1002/advs.202203296

**Published:** 2022-12-09

**Authors:** Binghui Jin, Yiling Yu, Chenghao Lou, Xiaodi Zhang, Bowen Gong, Jinghao Chen, Xiangxiang Chen, Zihan Zhou, Liqun Zhang, Jian Xiao, Jiajia Xue

**Affiliations:** ^1^ Beijing Laboratory of Biomedical Materials State Key Laboratory of Organic–Inorganic Composites Beijing University of Chemical Technology Beijing 100029 P. R. China; ^2^ Oujiang Laboratory School of Pharmaceutical Sciences Wenzhou Medical University Wenzhou 325035 China

**Keywords:** controlled release, electrospun nanofibers, gradient scaffolds, nerve guidance conduits, peripheral nerve repair

## Abstract

Peripheral nerve injury is a serious medical problem with limited surgical and clinical treatment options. It is of great significance to integrate multiple guidance cues in one platform of nerve guidance conduits (NGCs) to promote axonal elongation and functional recovery. Here, a multi‐functional NGC is constructed to promote nerve regeneration by combining ordered topological structure, density gradient of biomacromolecular nanoparticles, and controlled delivery of biological effectors to provide the topographical, haptotactic, and biological cues, respectively. On the surface of aligned polycaprolactone nanofibers, a density gradient of bioactive nanoparticles capable of delivering recombinant human acidic fibroblast growth factor is deposited. On the graded scaffold, the proliferation of Schwann cells is promoted, and the directional extension of neurites from both PC12 cells and dorsal root ganglions is improved in the direction of increasing particle density. After being implanted in vivo for 6 and 12 weeks to repair a 10‐mm rat sciatic nerve defect, the NGC promotes axonal elongation and remyelination, achieving the regeneration of the nerve not only in anatomical structure but also in functional recovery. Taken together, the NGC provides a favorable microenvironment for peripheral nerve regeneration and holds great promise for realizing nerve repair with an efficacy close to autograft.

## Graphical abstract

A multi‐functional electrospun fiber‐based NGC is constructed to promote nerve regeneration by combining ordered topological structure, density gradient of biomacromolecular nanoparticles, and controlled delivery of biological effectors to provide the topographical, haptotactic, and biological cues, respectively.

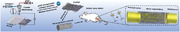

## 1 Introduction

Peripheral nerve injury (PNI) is a clinical disease that can generate motor and sensory impairment as well as significant long‐term disability,^[^ ^1^ ^]^ greatly affecting the life quality of patients and impairing their physical and mental health.^[^ ^2^ ^]^ Although nerve autograft has been known as the gold standard for clinical PNI repair, barely 50% of patients recover from the trauma, and it is difficult to repair a thick nerve in a large defect by autograft.^[^ ^3^ ^]^ As an alternative, tissue‐engineered nerve guidance conduits (NGCs) have shown great potential for nerve repair; they can ameliorate defects of autografts such as donor damage and inadequate sources.^[^ ^4^ ^]^ Several Food and Drug Administration (FDA) approved NGCs, such as NeuroMatrix, NeuroWrap, and Neurotube, have been used clinically. With the development of the NGC design, the treatment therapeutic has been improved. However, the outcomes are still limited compared to the autograft because of the design of the NGC, which stems mainly from the defect size and conduits failing to provide the necessary cues to guide axons and Schwann cells across very large defects.^[^ ^5^ ^]^ Therefore, it is of great importance and a great challenge as well to develop biologically active NGCs for simultaneously providing multiple induction signals, aiming to improve the microenvironment at the site of injury and promote axonal elongation and functional recovery.^[^ ^4^, ^6^ ^]^ Currently, various induction signals can be incorporated in scaffolds by designing their structure or morphology as well as loading growth factors and exogenous cells. These induction signals will promote the migration of endogenous cells as well as the regeneration and extension of axons, ultimately realizing nerve regeneration and functional recovery.

Owing to the capability of mimicking the structure and composition of extracellular matrix (ECM) and combining topographic and haptotactic cues into one scaffold, electrospun nanofibers have been extensively explored for tissue repair and regeneration.^[^ ^7^ ^]^ The main component of ECM is collagen, but the pristine electrospun collagen nanofibers can be limited by the mechanical strength and the toxicity of crosslinking agents. Endowing physical and biological cues to nanofiber‐based NGCs can improve peripheral nerve regeneration.^[^ ^8^ ^]^ Polycaprolactone (PCL) has received approval as a resorbable device for peripheral nerve repair and gained considerable interest in the research field due to its ease of fabrication and low processing costs.^[^ ^9^ ^]^ Its high processibility is attributed to the fact that PCL is highly soluble in a wide range of organic solvents and its crystalline nature enables easy formability at relatively low temperatures. With PCL, it is also easy to prepare nanofibers with electrospinning. Further, with the advantages of remarkable biocompatibility, biodegradability, and mechanical strength,^[^ ^10^ ^]^ uniaxially aligned PCL nanofibers could guide Schwann cells (SCs) migration and directional neurites extension of chick embryo dorsal root ganglion (DRG), promoting the peripheral nerve regeneration.^[^ ^8^, ^9^, ^10^, ^11^ ^]^ In addition to the topographical cue, the nervous system can respond to haptotactic cues, during which cell movement and neurites extension can be guided by the gradient of biomolecules.^[^ ^11^, ^12^ ^]^ Specifically, particles made of collagen, one of the main components of ECM, could be deposited in a density gradient profile on the uniaxially aligned fibers, allowing neurites to extend from PC12 cells and DRG along the direction of increasing particle density.

In addition to the topographical and haptotactic guidance, bioactive cues also play a significant role in promoting axonal elongation.^[^ ^13^ ^]^ Encapsulating repair‐promoting growth factors in the NGC and enabling their controlled release can enhance the efficacy of peripherical nerve repair.^[^ ^13^ ^]^ Typically, recombinant human acidic fibroblast growth factor (aFGF) has been discovered as a signaling molecule involved in axon growth and survival of neurons as well as in alleviating neuropathic injury.^[^ ^14^ ^]^ However, the practical therapeutic efficacy of aFGF is often limited due to its short half‐life and instability. In this case, a controllable release of aFGF will be beneficial for retaining its stability and sustainability by providing cues for nerve regeneration; and thus, improving the therapeutic efficacy.^[^ ^15^ ^]^ As an electrostatic fabrication method, coaxial electrospraying has been developed to fabricate uniform core–shell micro‐ or nanoparticles for the delivery of biological effectors or growth factors, which can also be easily combined with the electrospinning technique.^[^ ^16^ ^]^ In this case, biomacromolecular nanoparticles produced by coaxial electrospraying can protect the growth factors and prolong the storage time of biomacromolecules.^[^ ^16^, ^17^ ^]^ For now, in the design of NGC, it remains a challenge to simultaneously integrate multiple kinds of guidance cues into one scaffold by an efficient and simple method. Therefore, it will be of great worth to demonstrate the possibility of loading aFGF in the electrosprayed particles for controlled release in combination with electrospun fibers and the graded structure to construct a multi‐functional NGC.

**Figure 1:**
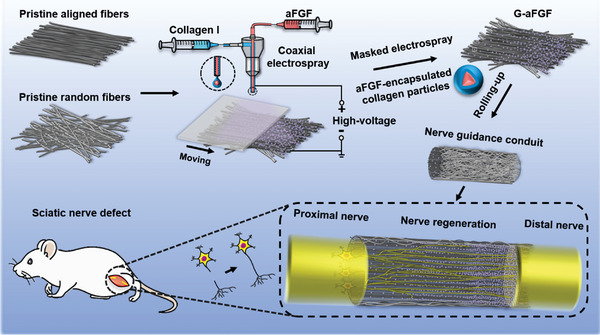
A schematic diagram showing the process for fabricating a graded‐NGC, which could combine the density gradient of aFGF‐encapsulated collagen particles with uniaxially aligned electrospun nanofibers to repair PNI.

Here, we applied a simple strategy to deposit biological effectors‐encapsulated biomacromolecular nanoparticles in a density gradient on the inner uniaxially aligned electrospun nanofibers for constructing an NGC with multiple guidance cues for the repair of PNI (**Figure** **1**). In combination with coaxial electrospraying and a movable plastic mask, aFGF‐encapsulated collagen particles were deposited on the uniaxially aligned fibers in a gradient density to fabricate the graded scaffold. We then investigated the release of aFGF and the effect of the scaffold on the proliferation of SCs, as well as on the neurites’ extension from both PC12 cells and DRG in vitro. Furthermore, the efficacy of the conduit constructed from the graded scaffold on the nerve regeneration, remyelination, and functional recovery was also demonstrated in a 10‐mm Sprague–Dawley (SD) rat sciatic nerve defect model in comparison with the autograft and uniform scaffold. The roles and mechanisms of the different guidance cues in the conduit on the peripheral nerve repair were then demonstrated.

## 2 Results and Discussion

During electrospinning, the rotating speed of the rolling collector was tuned to obtain a nanofiber mat with one layer composed of random nanofibers and another layer of uniaxially aligned nanofibers. The pristine aligned and random nanofibers showed smooth surfaces with an average diameter of 393 ± 127 and 493 ± 191 nm, respectively (Figure S1, Supporting Information). The orientation of the pristine random nanofibers was randomly distributed, while that of the uniaxially aligned nanofibers was mostly distributed in a certain narrow range of angles (Figure S2, Supporting Information), indicating that the uniaxially aligned nanofibers were highly oriented.

**Figure 2:**
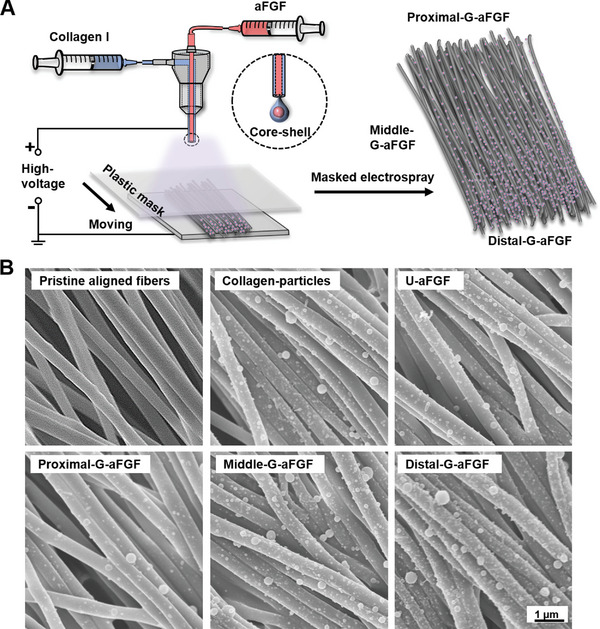
A) A schematic indicating the fabrication of the graded scaffold integrated with the density gradient of aFGF‐encapsulated collagen particles together with uniaxially aligned electrospun nanofibers via a masked coaxial electrospraying method. The plastic mask moves to vary the collection duration of the particles along the fiber alignment. B) SEM images of the different scaffolds.

In addition to the topographical cue, the introduction of biochemical cues is also essential for peripheral nerve repair.^[^ ^18^ ^]^ We first investigated the influence of the concentrations of aFGF on the proliferation of rat Schwann cells (RSC96 cell line) to determine the encapsulation amount of aFGF on the scaffold. As shown in Figure S3, Supporting Information, after incubation for 5 days, the proliferation of SCs was significantly promoted by increasing the concentration of aFGF to 50 ng mL^−1^ (*p <* 0.001). Afterward, the surface of the layer composed of uniaxially aligned nanofibers was further deposited with aFGF‐encapsulated collagen particles by a masked electrospray method (**Figure** **2**A). A movable mask was placed above the nanofiber mat and moved at a rate of 0.1 cm min^−1^ to vary the deposition time of particles, and the as‐obtained scaffold was labeled as G‐aFGF. The G‐aFGF scaffold was then divided into three sections with the same area and labeled as Proximal‐G‐aFGF, Middle‐G‐aFGF, and Distal‐G‐aFGF, respectively. The nanofiber mats with the aligned nanofibers deposited with pristine collagen particles (Collagen‐particles) or with aFGF‐encapsulated collagen particles (U‐aFGF) in a uniform density were also fabricated to serve as control groups, and the particle deposition density was controlled to be the same as that of Middle‐G‐aFGF. Figure 2B shows the scanning electron microscopy (SEM) images of the different scaffolds. From the proximal to distal position of the G‐aFGF scaffold, aFGF‐encapsulated collagen particles were increased. The collagen particles were all adhered well onto the surface of the nanofibers, and the average diameters of the pristine collagen particles and the aFGF‐encapsulated collagen particles in a uniform or graded distribution were 59 ± 9, 64 ± 7, and 71 ± 22 nm, respectively. During the co‐axial electrospraying process, the aFGF was encapsulated inside the collagen sheath.

To verify those aFGF‐encapsulated collagen particles, we used fluorescein isothiocyanate labeled bovine serum albumin (FITC‐BSA) as a model of aFGF and observed the scaffolds under a confocal laser scanning microscope (CLSM). As shown in Figure S4, Supporting Information, the pristine nanofibers were deposited with FITC‐encapsulated collagen particles, and the relative fluorescence intensity of Distal‐G‐FITC‐BSA was the highest compared to others (*p* < 0.001), demonstrating the increased density of the deposited particles from the proximal to distal position of the graded scaffold.

The water contact angles of Proximal‐G‐aFGF, Middle‐G‐aFGF, and Distal‐G‐aFGF were 111 ± 14°, 73 ± 2°, and 61 ± 5°, respectively (Figure S5A,B, Supporting Information). Collagen is a hydrophilic natural polymer; and thus, the deposition of collagen on the PCL nanofibers improved the hydrophilicity of the scaffold. As the density of particles increased, the water contact angle of the G‐aFGF scaffold was gradually reduced from the proximal to the distal position. After plasma treatment, the water contact angles of three sections of G‐aFGF were decreased from 37 ± 1° to 26 ± 2° and 17 ± 1°, respectively, from the proximal to a distal position (Figure S5A,B, Supporting Information). The superior hydrophilicity provided more beneficial conditions for cell adhesion and proliferation.

**Figure 3:**
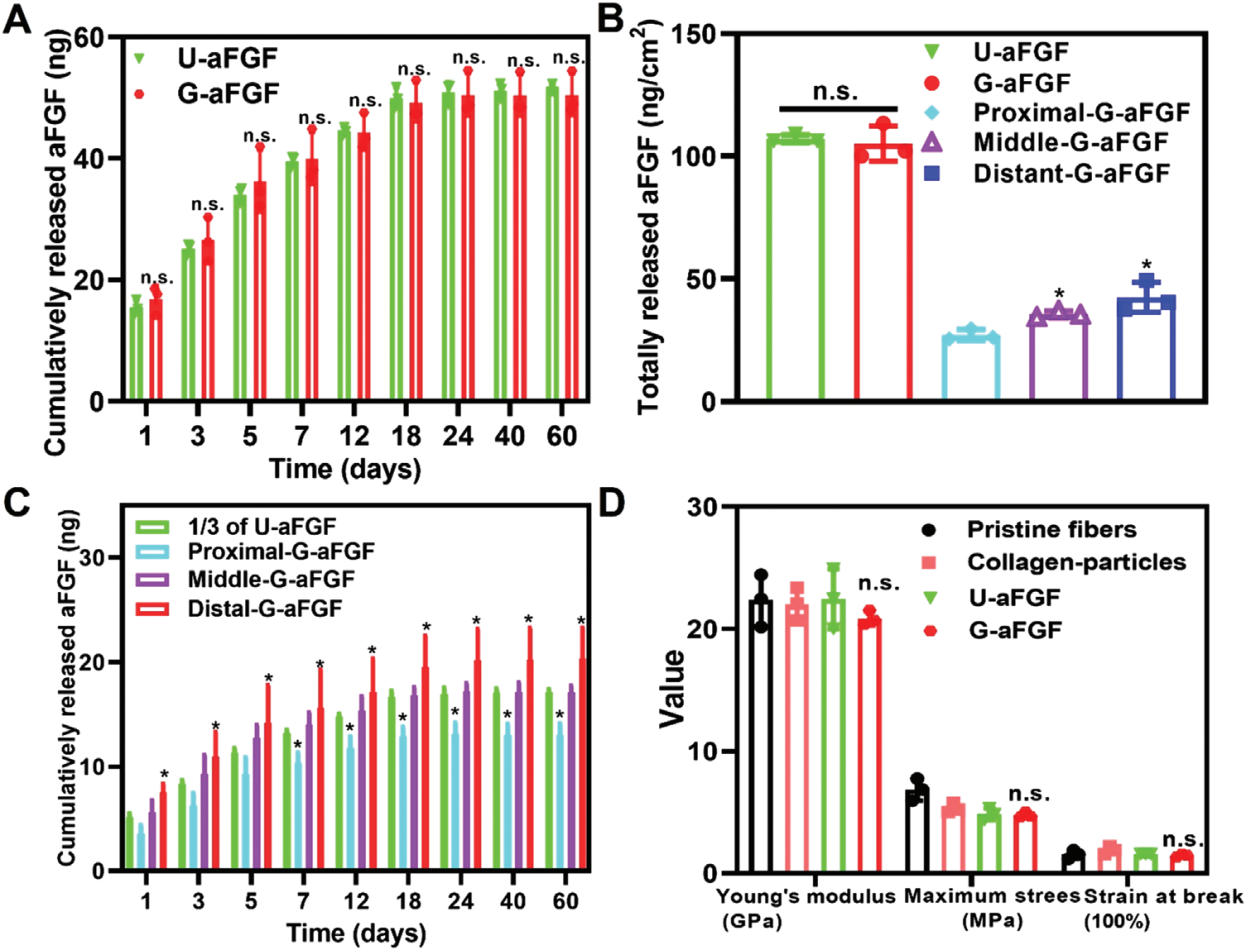
A) Cumulatively released mass of aFGF from the U‐aFGF and G‐aFGF scaffolds after 0, 1, 3, 5, 7, 12, 18, 24, 40, and 60 days. B) The total released mass of aFGF from the U‐aFGF, the three regions of G‐aFGF, and G‐aFGF after 60 days, respectively. *p < 0.05 (*n* = 3) in comparison to the group of Proximal‐G‐aFGF. ^#^ *p* < 0.05 (*n* = 3) in comparison to the group of 1/3 of U‐aFGF. C) Cumulatively released mass of aFGF in the three sections of G‐aFGF and 1/3 of U‐aFGF after 0, 1, 3, 5, 7, 12, 18, 24, 40, and 60 days. *p < 0.05 (*n* = 3) in comparison to 1/3 of U‐aFGF. D) The mechanical properties of the different scaffolds.

The release profiles of aFGF from the different scaffolds were then investigated. The aFGF‐encapsulated collagen particles could be released through diffusion and the degradation of collagen. **Figure** **3**A; Figure S6, Supporting Information show the release profiles of aFGF from the U‐aFGF and G‐aFGF scaffolds, indicating a slow release within 18 days. As the total deposited number of aFGF‐encapsulated collagen particles on the surface of the U‐aFGF and G‐aFGF scaffolds had no significant difference by controlling the deposition duration, the cumulatively released mass of aFGF from the two scaffolds had no significant difference as well after release for different periods. As shown in Figure 3B, the total released mass of aFGF from the U‐aFGF and G‐aFGF scaffolds with an area of 0.48 cm^2^ were 51 ± 1 and 50 ± 3 ng, respectively, which means that the loading contents of aFGF on the two scaffolds were 107 ± 1 and 105 ± 3 ng cm^−2^, respectively. Furthermore, the released mass of aFGF from the Distal‐G‐aFGF was greater than that from Middle‐G‐aFGF, both of which were greater than that from the Proximal‐G‐aFGF section (*p* < 0.05), indicating the loaded and released amounts of aFGF were increased with the increase of the particle's density. U‐aFGF was also divided into three regions with equal areas. As the deposition of aFGF‐encapsulated collagen particles in the three regions was similar, each fraction could be considered as 1/3 of U‐aFGF. After 1, 3, 5, 7, 12, 18, 24, 40, and 60 days, the cumulative release of aFGF in Distal‐G‐aFGF was greater than that of 1/3 of U‐aFGF (Figure 3C). The cumulative release of aFGF in the three sections of G‐aFGF maintained an increasing trend after different periods.

The mechanical properties of the different scaffolds were tested. As shown in Figure 3D, there were no significant differences in Young's modulus for the scaffolds consisting of Pristine fibers, Collagen‐particles, U‐aFGF, and G‐aFGF groups, as well as on the maximum stress and strain at break. These results demonstrated that the deposition of the biomacromolecular nanoparticles did not affect the excellent mechanical properties of the scaffolds, which could be applied as the NGC.

The proliferation of SCs plays a significant role in promoting nerve repair. As shown in Figure S7, Supporting Information, the proliferation of RSC96 cells on the aFGF scaffold was significantly promoted in comparison with that on the other scaffolds after incubation for 1, 3, and 5 days (*p* < 0.001), indicating that the proliferation of RSC96 cells was promoted by the deposition of collagen particles and the sustained release of aFGF. In addition, the superior effect of SCs proliferation in the G‐aFGF scaffold was due to the different amounts of released aFGF in the proximal, middle, and distal parts.

**Figure 4:**
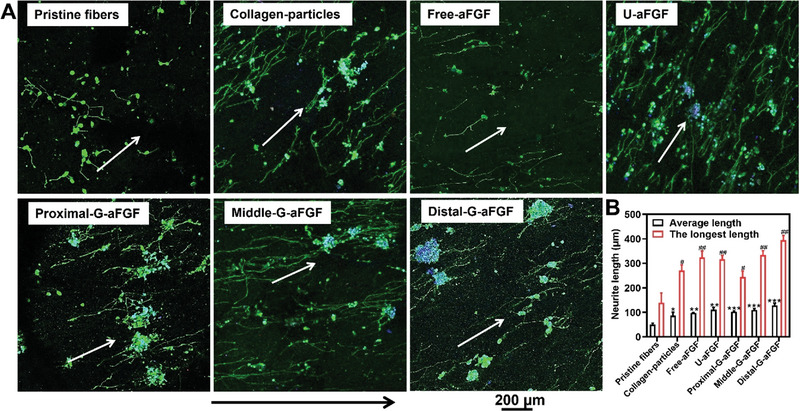
A) Fluorescence microscopy images of neurites extending from PC12 cells on the different scaffolds. The neurites and cell nuclei were stained with neurofilament‐200 (NF‐200, green) and DAPI (blue), respectively. The white arrows show the direction of fiber alignment, and the black arrow shows the gradient density of aFGF‐encapsulated collagen particles from the proximal to distal position of the G‐aFGF scaffold. B) The average and the longest lengths of neurites extend from PC12 cells on the different scaffolds. *^#^p* < 0.05 and *^##^p* < 0.01 in comparison to the group of Pristine fibers. *p < 0.05, **p < 0.01, and ***p < 0.001 in comparison to the group of Pristine fibers.

The differentiation of PC12 cells was induced by nerve growth factor (NGF) to examine the role of topography, surface gradient, and growth factor release on the extension of neurites. As shown in **Figure** **4**A, when PC12 cells were seeded on the different scaffolds consisting of Pristine fiber, Collagen‐particles, Free‐aFGF, U‐FGF, Proximal‐G‐aFGF, Middle‐G‐aFGF, and Distal‐G‐aFGF, the average lengths of neurites were 51 ± 4, 101 ± 11, 110 ± 1, 127 ± 3, 75 ± 5, 110 ± 7, and 154 ± 7 µm, respectively, while the longest lengths of the neurites were 128 ± 33, 236 ± 19, 269 ± 21, 334 ± 13, 231 ± 20, 344 ± 14, and 516 ± 16 µm (Figure 4B). The length of neurites extending from PC12 cells on the Distal‐G‐aFGF was larger than that on the others. The results showed that the uniaxially aligned topography, particles with surface gradient, and controlled release of aFGF could play a synergistic role in jointly promoting the extension of neurites.

**Figure 5:**
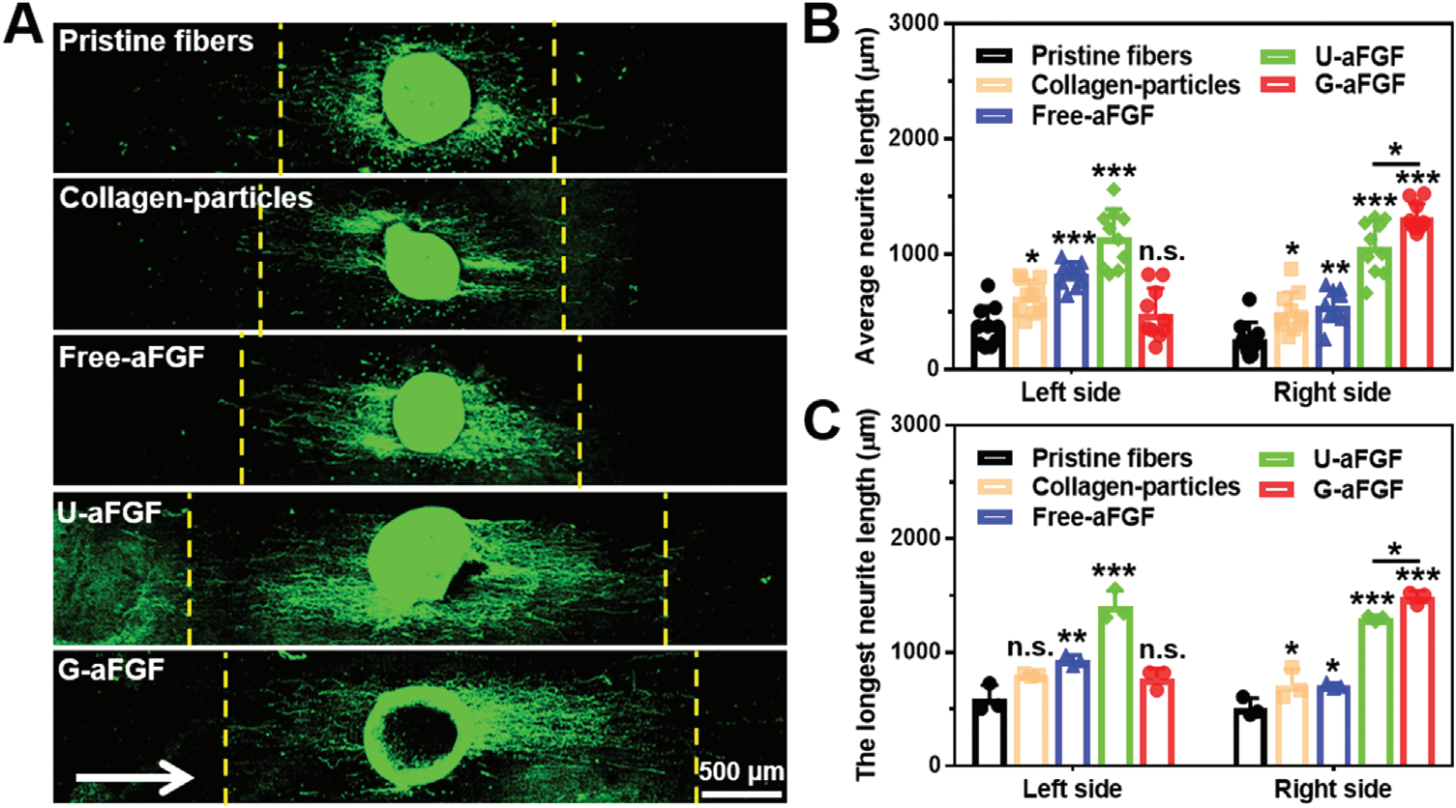
A) The fluorescence microscopy images of the typical extension of neurites from DRGs after being cultured for 5 days on different scaffolds. The neurites were stained with beta III tubulin (green). The white arrow shows the direction of the increasing density of collagen from the proximal to the distal of the G‐aFGF scaffold. B) The average and C) the longest lengths of neurites extension of DRGs cultured on the different scaffolds, respectively. *p < 0.05, **p < 0.01, and ***p < 0.0001 (*n* = 3) in comparison to the group of Pristine fibers.

The extracellular matrix (ECM) shows the important biochemical and structural function in morphogenesis by supplying topographical features in the extracellular milieu and creating tissue boundaries. Growth cones at the leading ends of nerve axons can detect and respond to these specific guiding environmental stimuli throughout neuronal development, driving neuronal pathfinding.^[^ ^6^ ^]^ The extension of neurites from DRGs after being cultured for 5 days on the different scaffolds was detected. As shown in **Figure** **5**A, the neurites were extended from both sides of DRGs and oriented in all groups following the direction of fiber alignment. For the graded scaffold, the density of collagen particles was increased from left to right in the micrographs. The average lengths of the neurites extending from DRGs cultured on Pristine fibers, Collagen‐particles, Free‐aFGF, U‐aFGF, and G‐aFGF scaffolds were tested, and those from the left side were 375 ± 55, 632 ± 46, 827 ± 33, 1141 ± 79, and 482 ± 70 µm, respectively; while those from the right side were 263 ± 46, 497 ± 55, 548 ± 47, 1061 ± 73, and 1315 ± 39 µm, respectively (Figure 5B). In addition, the longest lengths of the neurites on the left in each group were 589 ± 70, 802 ± 8, 931 ± 27, 1404 ± 79, and 771 ± 51 µm, respectively, while those for the right side were 514 ± 48, 717 ± 79, 708 ± 15, 1300 ± 15, and 1483 ± 33 µm, respectively (Figure 5C). Therefore, with the incorporation of collagen particles, the extension of neurites was significantly promoted in comparison with pristine fibers. On aFGF‐encapsulated collagen particles, the extension of the neurites was further promoted, which showed a better performance than the group incorporating free aFGF, indicating that the released aFGF retained the bioactivity and a sustained release was beneficial for the neurite extension. When the bioactive particles were deposited in a gradient profile, the neurites were guided to extend in the direction of particle density increasing, demonstrating the haptotactic cue generated by the density gradient. Therefore, a multi‐functional electrospun fiber‐based scaffold was constructed to promote the directional extension of neurites by combining ordered topological structure, density gradient of biomacromolecular nanoparticles, and controlled delivery of biological effectors to provide the topographical, haptotactic, and biological cues, respectively. Meanwhile, as reported previously, the graded growth factor directed SCs migration and promoted axon attraction through several important signal pathways, involving Ras‐associated protein 1 (Rap1), cell adhesion molecules, and mitogen‐activated protein kinase (MAPK).^[^ ^11b^ ^]^ Thus, since the use of graded aFGF in this study, Schwann cells could also sense the contact guidance from the uniaxially aligned nanofibers and the gradient of the particles, and with the supplement of aFGF, the migration of Schwann cells from the DRG could be further accelerated on the G‐aFGF scaffold.^[^ ^7a^ ^]^

**Figure 6:**
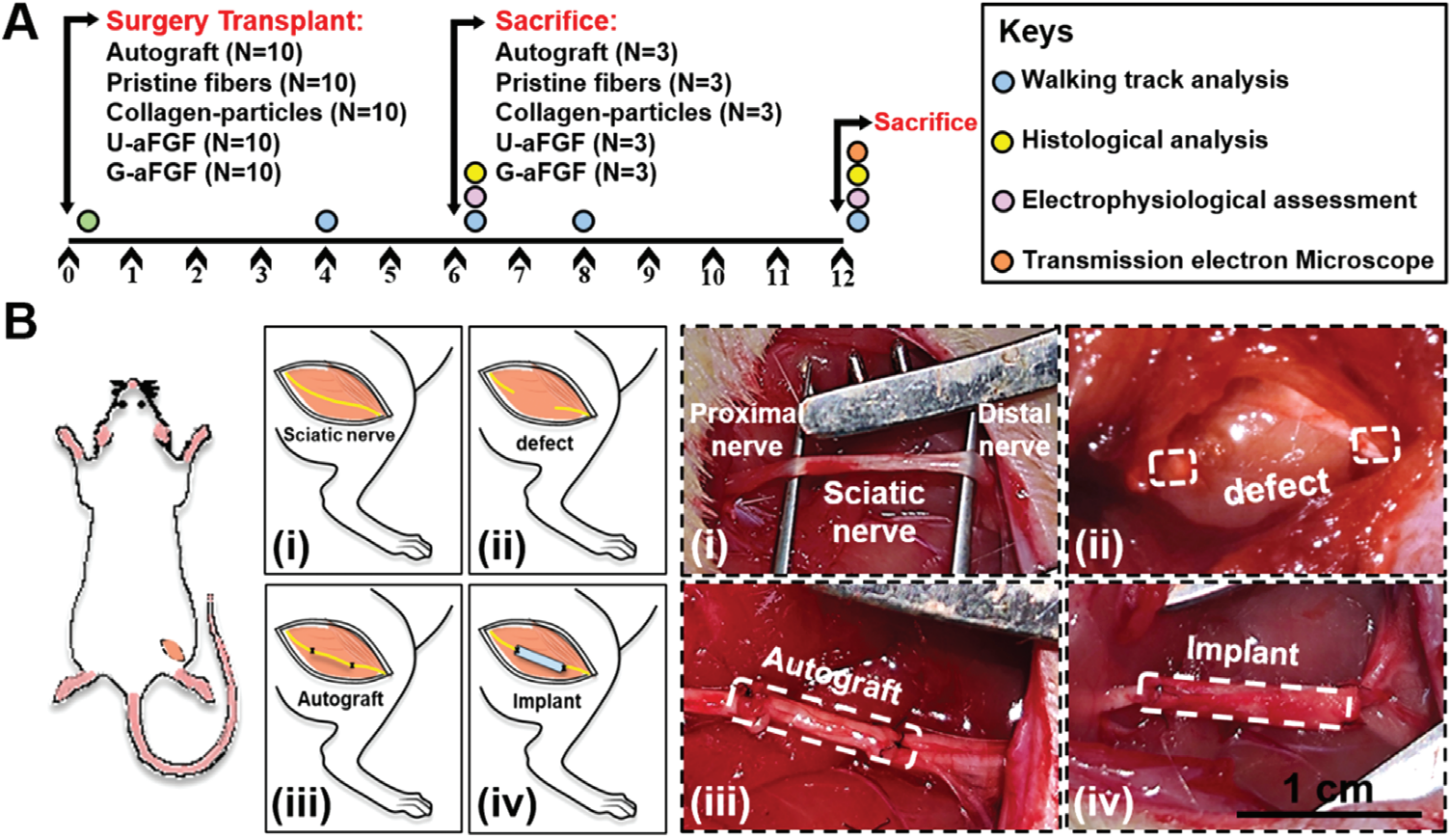
Animal surgery. A) Schematic of the experimental timeline and design. B) Illustrations and real images of B‐i) SD rat sciatic nerve, B‐ii) 10‐mm nerve defect, B‐iii) nerve autograft transplantation, and B‐iv) implant transplantation during the surgery.

Despite intense research efforts, the outcomes of current therapeutic treatment to PNI are still limited.^[^ ^5^ ^]^ It is of great importance and a great challenge as well to develop biologically active NGCs for both achieving structural guidance and simultaneously providing multiple induction signals.^[^ ^4^, ^6^ ^]^ The rat sciatic model with a gap of 10 mm has been the most commonly used for nerve regeneration study of synthetic scaffolds.^[^ ^19^ ^]^ Herein, the efficacy of the fiber‐based NGCs in peripheral nerve injury was evaluated in vivo by bridging a 1‐cm sciatic nerve defect in rats. NGC with a size of 1.5 mm in diameter and 1.2 cm in length was produced by rolling up the electrospun fiber mat (Figure S8, Supporting Information). Different types of NGCs were constructed from the scaffolds of Pristine fibers, Collagen‐particles, U‐aFGF, and G‐aFGF. During the surgery, the sciatic nerves of 50 male rats were defected and sutured with the two ends of the NGCs to leave an approximate 10 mm gap between the proximal and distal nerve segments. Autograft was applied as the control group, and all assessments were carried out at 6 and 12 weeks after surgery to give a better comparison (**Figure** **6**).

**Figure 7:**
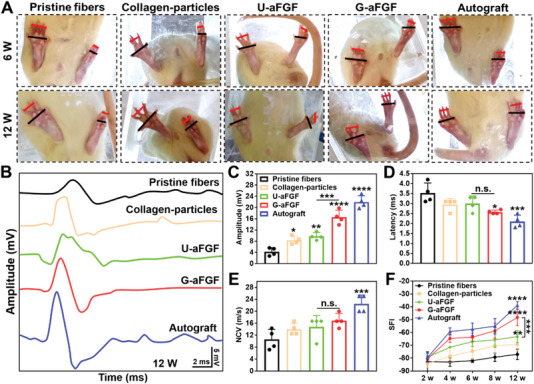
Functional recovery evaluation. A) Images of hind paws of the rats in five groups at 6 and 12 weeks. B) Electrophysiological assessment of CMAP. C) Quantitative analysis of CMAP amplitude. D) Quantitative analysis of CMAP latency. E) Nerve conduction velocity (NCV). F) Statistical analysis of SFI at different time points after surgery. *p < 0.05, **p < 0.01, ***p < 0.001, and ****p < 0.0001 (*n* = 4) in comparison to the group of Pristine fibers.

At 12 weeks following nerve injury, walking track analysis and electrophysiologic testing were performed to evaluate the functional recovery in five groups. Specifically, the amplitude of compound muscle action potential (CMAP) in the G‐aFGF group was considerably higher than that in the Pristine fibers, Collagen‐particles, and U‐aFGF groups (**Figure** **7**B,C). In addition, except for the autograft group, the G‐aFGF group had a shorter conduction delay and a higher conduction velocity than the other three treatment groups using the fiber conduits (Figure 7D,E). Photographs of rat hind paws, as well as quantitative data of sciatic function index (SFI), revealed that the G‐aFGF group recovered the best among the four fiber‐based conduit groups, while the impact was still inferior to autografts (Figure 7A,F).

**Figure 8:**
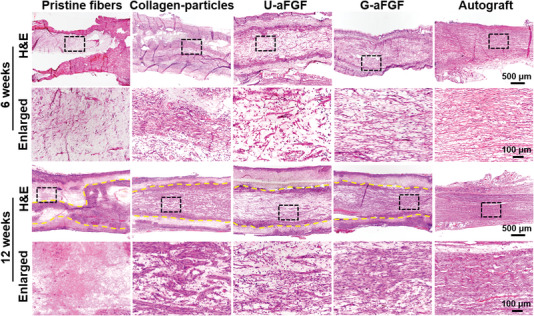
After surgery, H&E staining images of the longitudinal slices of regenerated nerve in five groups at 6 and 12 weeks, respectively. Dotted yellow lines indicate the edge between the scaffold and the regenerated tissue.

**Figure 9:**
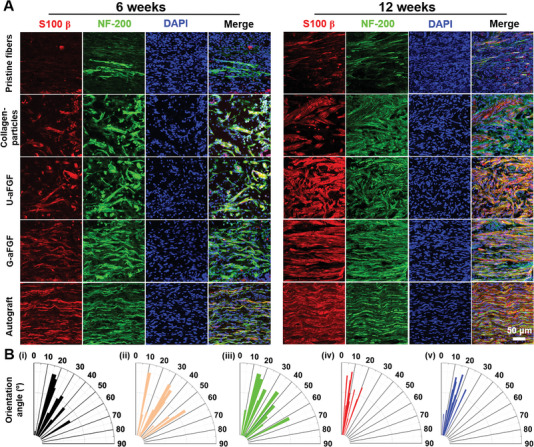
A) After surgery, immunofluorescent images of the longitudinal slices of regenerated nerve in five groups at 6 and 12 weeks, respectively. SCs (S100*β*), axons (NF‐200), and nuclei were stained, respectively. B) Orientation angles of nerve fibers on B‐i) Pristine fibers, B‐ii) Collagen‐particles, B‐iii) U‐aFGF, B‐iv) G‐aFGF, and B‐v) Autograft groups at 12 weeks.

PCL received FDA approval for peripheral nerve repair in 2005 with its remarkable biocompatibility, biodegradability, and mechanical strength. However, the results of pre‐clinical studies were unsatisfactory and there were only some myelinated nerve fibers evident after 12 weeks of implantation in a 10 mm nerve gap defect.^[^ ^8^ ^]^ At 6 and 12 weeks, the regenerated tissue sections in five groups were performed with histological analysis to observe the effect of different types of NGCs on nerve repair. As illustrated in **Figure** **8**, hematoxylin–eosin (H&E) staining of longitudinal slices of five groups revealed that the Pristine fibers group had minimal axon outgrowth and nerve cell infiltration, while the other three groups showed clear nerve tissue expansion. Despite the slow rate of degradation of PCL (Figure 8, dotted yellow lines) in all four groups, the lower inflammatory effect was also detected by the immunostaining of CD68 (macrophage marker) in Figure S9, Supporting Information. In addition, nerve fibers in the G‐aFGF group were more ordered than those in the Collagen‐particles and U‐aFGF groups. These results were further confirmed by the immunostaining of the S100 beta (SCs marker) and NF‐200 (axon marker) of sections. As shown in **Figure** **9**, more SCs migration and longer axon elongation were induced by the deposition of gradient collagen particles on the aligned nanofibers in the G‐aFGF group. Further, nerve fibers in G‐aFGF had orientation angles from 0° to 25°, while it was 0° to 58°, 0° to 55°, and 0° to 60° for Pristine fibers, Collagen‐particles, and U‐aFGF groups, implying that the multiple guidance cues play an integrated role in directing nerve regeneration after nerve injury.

**Figure 10:**
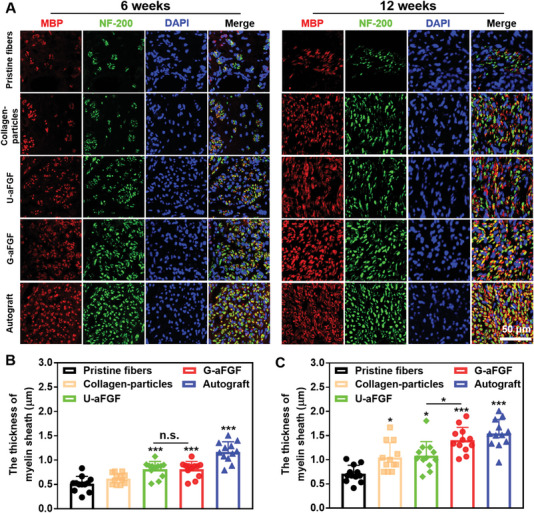
A) After surgery, immunofluorescent images of the cross‐slices of the regenerated nerve in five groups at 6 and 12 weeks, respectively. Myelin sheath, axons, and nuclei were stained, respectively. B,C) Statistical analysis of the thickness of myelin sheath for five groups at 6 and 12 weeks, respectively. *p < 0.05, **p < 0.01, and ***p < 0.0001 (*n* = 4) in comparison to the group of Pristine fibers.

The myelin sheath, which is made up of SCs that wrap tightly around the axon in the peripheral nervous system, plays an important role in nerve signal conduction.^[^ ^20^ ^]^ Therefore, the cross‐sections of regenerated tissues were double immune‐stained with myelin basic protein (MBP) and NF‐200 to monitor the remyelination in each group at 6 and 12 weeks, respectively. As shown in **Figure** **10**, MBP and NF‐200 expression were increased in all five groups at 12 weeks compared to at 6 weeks, indicating that all groups went through continuous remyelination during the 12 weeks. It is worth noting that this result was consistent with the previous SCs migration and axon elongation data which verified the excellent induction of the gradient collagen particles further.

**Figure 11:**
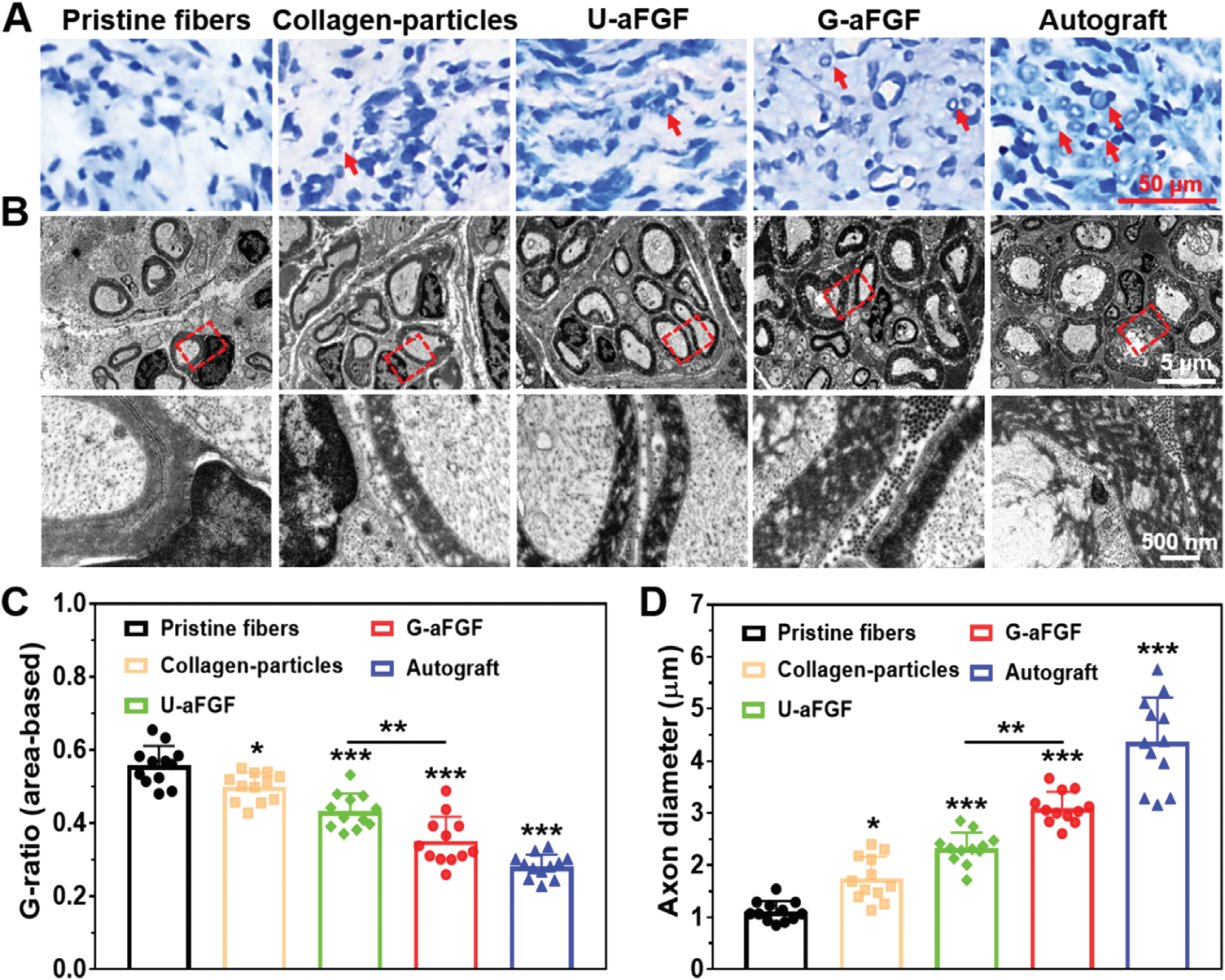
After surgery, the histology and electron microscopy assessment of myelin in the regenerated nerves in five groups at 12 weeks. A) Images of TB staining of the cross‐slices of regenerated nerves. B) TEM images of regenerated nerves. C) Statistical analysis of G‐ratio for five groups. D) Statistical analysis of axon diameter for five groups. *p < 0.05, **p < 0.01, and ***p < 0.0001 (*n* = 4) in comparison to the group of Pristine fibers.

For further validation, toluidine blue (TB) staining of the cross‐slices (**Figure** **11**A) and transmission electron microscopy (TEM) observation of the regenerated tissues (Figure 11B) were performed. Compared to the Pristine fibers group, the densities of myelinated axons were significantly increased in the Collagen‐particles, U‐aFGF, G‐aFGF, and autograft groups. Moreover, the density was considerably greater in the G‐aFGF group than in the other two fiber‐based conduits treatment groups. In addition, TEM studies of nerve fiber microstructure showed that the combination of gradient particles and aFGF together with aligned pristine fibers resulted in decently regenerated nerves composed of thick and well‐organized myelinated fibers. Furthermore, from Figure 11C,D, statistical analysis indicated that compared to the Pristine fibers, Collagen‐particles, and U‐aFGF groups, the G‐aFGF group showed a significantly lower G‐ratio (the area of the inner axon/the area of outer myelinated fiber) and considerably larger axon diameter.

**Figure 12:**
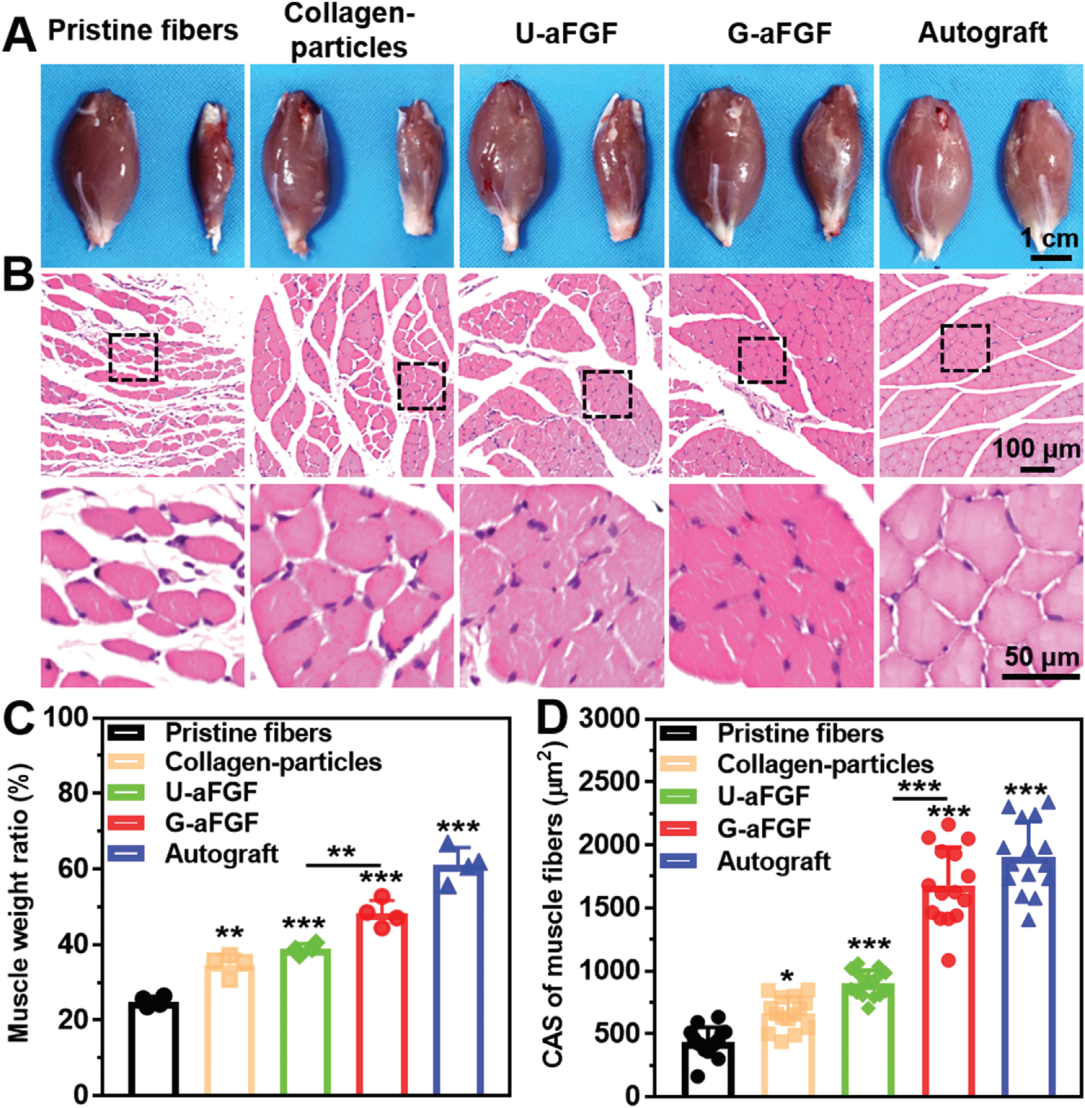
Morphology and histology of gastrocnemius muscles at 12 weeks post‐surgery. A) Representative images of the muscles of the normal (left) and experimental (right) sides. B) H&E staining images of the cross‐slices of muscles from the experimental side. C) Quantitative analysis of the muscle wet weight ratio from the experimental side. D) Quantitative analysis of cross‐section area of muscle fibers from the experimental side. *p < 0.05, **p < 0.01, and ***p < 0.0001 (*n* = 4) in comparison to the group of Pristine fibers.

Physiologically, the gastrocnemius muscle is the end target tissue innervated by the sciatic nerve.^[^ ^21^ ^]^ Hence, examinations of the weight and atrophy of gastrocnemius can indirectly represent reinnervation during nerve repair (**Figure** **12**). At 12 weeks post‐surgery, the bilateral gastrocnemius of rats in five groups was isolated, photographed, and weighed (Figure 12A). When compared to the Pristine fibers group, the shape of muscles in Collagen‐particles, U‐aFGF, and G‐aFGF groups suggested that the deposition of particles and release of aFGF could significantly improve muscle recovery to varying degrees. In addition, as shown in Figure 12C, the wet weight ratio in the G‐aFGF group was higher compared to that in the Collagen‐particles and U‐aFGF groups, confirming an additional benefit of using the gradient particle deposition and aFGF release for muscle repair. From Figure 12B,D, further staining of gastrocnemius cross‐sections and measurement of muscle fiber area gave a similar result to that of gastrocnemius weight.

From the anatomical structure of the repaired nerve, we could observe that the performance of the G‐aFGF treatment group approximated that of the autograft. From the CMAP evaluation and target muscle function, the graded scaffold did not match autograft in terms of functional recovery. The function of the peripheral nerve can be determined to a large extent by the myelin sheath that allows nerve impulses to travel rapidly and effectively. To further improve the efficacy of the G‐aFGF group, a cellular component such as differentiated stem cells or Schwann cells can be incorporated into the conduit. In addition, during the nerve repair, the combined use of different types of growth factors will also be a good way to further improve the cell migration and proliferation to improve the regeneration and formation of myelin sheath for promoting functional recovery.

## 3 Conclusion

PNI is a serious medical challenge with limited surgical and clinical treatment options, and the concept of replacing surgical nerve autografts with NGCs has always attracted the attention of the materials and medical community. To replace nerve autografts and further promote peripheral nerve repair, we constructed a multifunctional NGC integrated with uniaxially aligned fibers and density gradient of aFGF‐encapsulated collagen particles together, which accomplished controlled delivery of aFGF and provided the topographical, haptotactic, and biological cues, respectively. Electrospun nanofibers scaffold promoted the proliferation of RSC96 cells and facilitated the neurite's extension from both PC12 cells and primary DRGs in vitro. In addition, NGC based on the graded nanofiber scaffold improved the axon elongation in repairing a 1‐cm sciatic nerve defect and promoted remyelination, reinnervation, and functional recovery. Taken together, we constructed fiber‐based NGCs that integrated multiple regeneration‐inducing signals to offer a favorable microenvironment for the repair of PNI, which holds significant potential for the repair of long‐segment nerve defects.

## 4 Experimental Section

### Materials

PCL (*M*_w_ = 80 000 g mol^−1^), dichloromethane, formaldehyde, *N*,*N*‐dimethylformamide, and glacial acetic acid were purchased from Sigma–Aldrich. Type I collagen was purchased from Solarbio Life Sciences, Beijing, China. DMEM and phosphate‐buffered saline (PBS, pH 7.0–7.2) in 0.1 µm Sterile Filtered were purchased from Hyclone. Fetal Bovine Serum of heat inactivation (FBS) and Hank's buffered salt solution (HBSS) were purchased from Thermo Fisher Scientific. The antibodies were purchased from either Abcam or Thermo Fisher. aFGF was gained from Wenzhou Medical University, China. PC12 cells, poly‐*L*‐Lysin (PLL), and PC12 cell culture medium were purchased from Procell Life Science & Technology Co., Ltd, China.

### Electrospinning of Pristine PCL Nanofibers

The electrospinning technique was applied to fabricate PCL nanofibers. PCL was dissolved in *N*,*N*‐dimethylformamide and dichloromethane mixture (1:4) to prepare a homogeneous electrospinning PCL solution (12 wt%), followed by injecting at a rate of 0.0167 mL min^−1^ through an 18‐gauge blunt needle; and then, a high voltage of 12 kV was applied. Aluminum foil was covered on the roller and used to collect the electrospun nanofibers. The rotating speeds of the roller were changed from 1000 to 800, 600, 400, and 200 rpm every 2 h, and the electrospinning process was terminated after 10 h. The top layer collected at the low rotating speed was composed of pristine random fibers, while the bottom layer directly in contact with the aluminum foil was composed of uniaxially pristine aligned nanofibers.

### Deposition of Payload‐Encapsulated Particles on the PCL Nanofiber Mat

The aFGF‐encapsulated collagen particles were deposited on the nanofiber mat (length of 1.2 and 0.5 cm in width) in a density gradient by masked, coaxial electrospray method. Type I collagen was dissolved in 70% acetic acid aqueous solution to prepare 20 mg mL^−1^ collagen solution, followed by applying as the outer solution for coaxial electrospraying, while 0.5 mg mL^−1^ aFGF solution was applied as the inner solution. For the masked‐electrospraying setup, a movable mask was attached to a third pump and placed 2 cm above the fiber mat with the uniaxial aligned nanofibers upside. During the co‐axial electrospraying process, the outer collagen solution and inner aFGF solution were pumped out at a rate of 0.5 and 1.5 mL h^−1^ by a coaxial blunt needle, respectively, and a high voltage of ≈18 kV was applied. The aFGF‐encapsulated collagen particles were first deposited on the surface of aligned fibers for 2 min. Afterward, the plastic mask was moved at a speed of 0.1 cm min^−1^ and lasted for 10 min to generate a density gradient of particles on the surface of the nanofibers, achieving the graded scaffold of G‐aFGF. The obtained samples were treated with plasma for 20 s, followed by storing at −20 °C. As a comparison, Collagen‐particles and U‐aFGF scaffolds were also fabricated by depositing collagen particles and aFGF‐encapsulated collagen particles in a uniform distribution on the surface of the fiber mat for 7 min without the use of the plastic mask, respectively. To visualize the distribution of aFGF‐encapsulated collagen particles, FITC‐BSA‐encapsulated collagen particles were also deposited on the fiber mat in a uniform or gradient profile using a similar method by changing the inner aFGF solution to 0.5 mg mL^−1^ FITC‐BSA solution. Different scaffolds were observed under CLSM, and the relative fluorescence intensities were measured using Image J software.

### Release of aFGF from the Fiber Scaffolds

The fabricated G‐aFGF scaffold with a length of 1.2 and 0.4 cm in width was cut into three 4‐mm long segments with the same area along the fiber alignment, which were labeled as Proximal‐G‐aFGF, Middle‐G‐aFGF, and Distal‐G‐aFGF, respectively, along the direction of particles density increasing. The U‐aFGF scaffold was also cut into three total same 4‐mm long segments with the same area along the fiber alignment (1/3 of U‐aFGF). The 1/3 of U‐aFGF, Proximal‐G‐aFGF, Middle‐G‐aFGF, and Distal‐G‐aFGF samples were soaked in 1 mL of PBS. Then, all of the 1 mL suspension was replaced with fresh PBS and then stored at −20 °C after 1, 3, 5, 7, 12, 18, 24, 40, and 60 days. The aFGF ELISA kit was used to analyze the released concentration of aFGF, and then, the total and cumulatively released amounts of aFGF were calculated correspondingly.

### Mechanical Testing of the Different Scaffolds

Pristine fibers, collagen‐particles, U‐aFGF, and G‐aFGF were cut into parallel samples with a length of 30 mm and a width of 15 mm, and the thicknesses of different samples were measured by digital calipers with a precision of 0.01 mm. The Young's modulus, maximum stress, and strain at the break of different scaffolds were tested by a tensile machine and obtained from triplicate samples in each group.

### Influence of aFGF on the Proliferation of Schwann Cells

The RSC96 cell line is a spontaneously transformed rat Schwann cell line which was purchased from Sciencell Research Laboratories and cultured in a medium consisting of DMEM, 10% FBS, and 1% Penicillin–Streptomycin solution. 5 × 10^3^ cells mL^−1^ RSC96 cells (1 mL per well) were seeded in the well of a 24‐well plate and then incubated with different concentrations of aFGF of 0, 10, 20, 30, 40, 50, 60, 70, 80, 90, and 100 ng mL^−1^. After incubation for 5 days, the optical densities of cells in the different groups were determined by cell counting kit‐8 according to the previous report.^[^ ^22^ ^]^

### The Proliferation of Schwann Cells on the Fiber Scaffolds

The proliferation of the RSC96 cells on the different scaffolds (i.e., Pristine fibers, Collagen‐particles, U‐aFGF, and G‐aFGF) was investigated. Briefly, the scaffolds were fixed in the wells of a 24‐well plate, sterilized under UV for 0.5 h, and then, the cells were seeded onto the scaffold and incubated in a 1 mL culture medium. After incubation for 1, 3, and 5 days, the optical densities of cells in the different groups were determined by cell counting kit‐8 assay according to the previous report, respectively.^[^ ^22^ ^]^ In addition, the influence of free‐aFGF on the proliferation of cells was also investigated, and this group served as the control group (Free‐aFGF). The cells were cultured on aligned fibers of the pristine PCL fiber mat in 1 mL of culture medium containing free‐aFGF solution at a concentration of 50 ng mL^−1^, which was equivalent to the amount of aFGF released from the scaffold. Triplicate samples in each group were used for the study.

### Neurites Extension from PC12 Cells Cultured on the Fiber Scaffolds

The different scaffolds were immersed in 0.1 mg mL^−1^ PLL solution at 4 °C overnight. PC12 cells were cultured in 1 mL of culture medium and then seeded at a density of 5 × 10^3^ cells per mL on the different scaffolds. Afterward, 50 ng mL^−1^ NGF was added to induce the differentiation and neurites extension from PC12 cells. After being cultured for 9 days, the different scaffolds were fixed in 3.7% formaldehyde at 25 °C for 30–50 min, followed by permeabilizing with 0.1% Triton X‐100 for 5–15 min. After being blocked with 3% BSA for 1 h, PC12 cells were stained with anti‐NF‐200 for 12 h at 4 °C, followed by combining with Alexa Fluor 488 goat anti‐mouse IgG (1:200) secondary antibody at 25 °C for 1 h; then, cell nuclei were stained with DAPI for 10 min. Fluorescence micrographs were captured by CLSM, and the longest and average lengths of neurites extending from the random 50 PC12 cells were measured by Image J software through the fluorescence micrographs.

### Isolation and Culture of DRGs

A total of 15 DRGs were isolated from the lumbar region of the spinal column in two postnatal day1 SD rats each time. After carefully cutting off attached nerve roots, 1 primary DRG was seeded onto the surface of the sample and cultured in a modified neurobasal medium consisting of 2% B27, 2 mmol L^−1^ L‐glutamine, and 1% Penicillin–Streptomycin. After culturing for 5 days, DRGs were fixed in 4% formaldehyde for 15 min and permeabilized for 15 min, followed by blocking with 5% BSA for 1 h, and then, incubating with beta III Tubulin (1:500) overnight. This was followed by detection using Alexa Fluor 488 donkey anti‐rabbit IgG (1:1000) secondary antibody at 25 °C for 1 h, and micrographs were captured using CLSM. The experiment was repeated three times and triplicate samples in each group were used for the study.

### Animal Surgery

All animal procedures followed the animal care guidelines of Wenzhou Medical University and were under the ethical approval of the Animal Experimental Committee of Wenzhou Medical University. A total of 50 healthy male SD rats (180 to 200 g) were purchased and randomly divided into five groups: Autograft, Pristine fibers, Collagen‐particles, U‐aFGF, and G‐aFGF. Briefly, rats were anesthetized with 1% sodium pentobarbital (0.3 mL per 100 g). After removing the hair from the right hind limb, male rats were fixed on an operating table in the prone position, and the skin of the surgical site was disinfected by an iodophor. Following the separation of skin and muscle, the sciatic nerve of the rat was exposed and sharply cut to create a 10‐mm defect. According to the divisions mentioned above, the nerve gaps were bridged with the reversed excised nerves for the autograft group and various NGCs for the other four groups by 8/0 nylon sutures. Last of all, all rats were intramuscularly injected with 800 000 units of penicillin to avoid infection after the muscle and skin were stitched by 4/0 silk sutures.

### Walking Track Analysis

Walking track analysis was conducted at 2, 4, 6, 8, and 12 weeks post‐surgery to examine the motor recovery in each group. Briefly, after staining the hind limbs with red ink, rats were forced to run through a 50 cm × 15 cm × 20 cm box where a piece of white paper was placed. By measuring the several parameters of rats’ footprints, the SFI was calculated based on the following formula: SFI = 109.5 × (ETS−NTS)/NTS − 38.3 × (EPL−NPL)/NPL + 13.3 × (EIT−NII)/NIT − 8.8. Toe spread (TS) is the distance between the 1st and 5th toes, inter‐toe spread (IT) is the distance between the 2nd and 4th toes, and PL is the length of the footprint. The E in the formula indicates the experimental or injured side while the N indicates the normal or uninjured side.

### Electrophysiological Assessment

At 6 and 12 weeks post‐surgery, rats were anesthetized, and the motor nerve function of the injured side was assessed by electromyography (Neuro Exam M‐800, medcomtech, China). Briefly, after putting the stimulating electrode on the proximal and distal positions of the male rats’ injured nerve and a recording electrode at the gastrocnemius muscle, the CMAPs of the rats in the different groups were recorded under a preset electrical stimulation (30 mA, 1 Hz). The latencies and peak amplitudes of CMAP were calculated correspondingly.

### Histological Analysis of Regenerated Nerve Tissues

At 6 and 12 weeks post‐surgery, SD rats were sacrificed, and nerves of the experimental side were obtained (*n* = 3 and *n* = 7 for each group at 6 and 12 weeks, respectively). After being fixed in freshly‐prepared 4% paraformaldehyde for 6 h, nerve tissues were immersed in 20% sucrose for 12 h. After being soaked in 30% sucrose for another 12 h, tissues were embedded in O.C.T Compound (4583, SAKURA, USA) and quickly frozen by liquid nitrogen. By using a cryostat microtome (CM1950, Leica, Germany), the samples were cut longitudinally into 10 µm slices and cut transversely from the distal end into 10 µm sections. For H&E staining and TB staining, longitudinal sections were fixed in methanol and washed with deionized (DI) water two times. Sections were then stained with Hematoxylin for 3.5 min and Eosin for 6 s or TB for 3 min. For immunofluorescent staining, both longitudinal and transverse sections were used. After being fixed in methanol and washed with PBS three times, sections were antigen retrieved using 0.25% trypsin at 37 °C, and then, incubated with 5% BSA at 37 °C for another 30 min. Next, longitudinal sections were double‐stained with chicken anti‐NF‐200 antibody (ab4680, Abcam, UK) and rabbit anti‐S100 beta antibody (ab40390, Abcam, UK) or rabbit anti‐CD68 antibody (DF7518, Affinity, USA) while transverse sections were stained with chicken anti‐NF‐200 antibody and rabbit anti‐MBP antibody (ab40390, Abcam, UK) at 4 °C. After 12 h, all sections were incubated with secondary antibodies goat anti‐chicken (ab150169, Abcam, UK) and donkey anti‐rabbit (ab6799, Abcam, UK) at 37 °C for 1 h; sections were mounted with DAPI Fluoromount‐G (Yeasen, China), followed by washing with PBS for three to five times. The images were captured under a light microscope (Nikon, Japan) and the fluorescence images were captured using CLSM (Ti‐E&A1 plus, Nikon, Japan).

### TEM Investigation of the Regenerated Nerve

At 12 weeks after surgery, the distal end of the regenerated nerves with the size of 1–2 mm^3^ in each group was collected and fixed in 2.5% glutaraldehyde solution overnight at 4 °C. Following post‐fixing in 1% osmium tetroxide solution and blocking with 1% uranyl acetate, specimens were then dehydrated and embedded in Araldite. For TEM observation, ultrathin sections (80 nm) of each sample were sliced. The TEM images were collected, and the average *g*‐ratio and the diameter of myelinated nerve fibers were measured.

### Gastrocnemius Muscle Evaluation

Rats were sacrificed 12 weeks post‐surgery, and both healthy and injured sides of gastrocnemius muscles were collected and weighed to calculate the weight ratio. Then, samples were immersed in a 4% paraformaldehyde solution for 24 h. After being dehydrated and embedded in paraffin, muscles were cut transversely to obtain 5 µm slices for H&E staining. The cross‐sectional area of muscle fibers was measured.

### Statistical Analysis

Image J software was applied for image processing, and Origin 2021 or GraphPad Prism was used for graphing and statistical analysis. Experimental data were presented as mean ± standard deviation and assessed with one‐way ANOVA. *p <* 0.05 was regarded as statistically significant.

## Conflict of Interest

The authors declare no conflict of interest.

## Author Contributions

Investigation, data curation, visualization, and writing‐original draft: B.J. Investigation, data curation, validation, and writing‐original draft: Y.Y. Validation and investigation: C.L., X.Z., and B.G. Resources and investigation: J.C. Investigation: X.C. and Z.Z. Conceptualization, methodology, and supervision: L.Z. Conceptualization, methodology, supervision, and review and editing: J. Xiao. Conceptualization, methodology, supervision, writing, and review and editing: J. Xue

## Supporting information

Supporting Information

## Data Availability

The data that support the findings of this study are available from the corresponding author upon reasonable request.
