# Supplementary material for: Combining a Density Gradient of Biomacromolecular Nanoparticles with Biological Effectors in an Electrospun Fiber‐Based Nerve Guidance Conduit to Promote Peripheral Nerve Repair
Source: Adv Sci (Weinh). 2022 Dec 9;10(4):2203296. doi: 10.1002/advs.202203296 (PMC9896046; doi:10.1002/advs.202203296)
Supplement: Supplementary file 1 — Supporting Information [file ADVS-10-2203296-s001.pdf]

Supporting Information

Combining A Density Gradient of Biomacromolecular Nanoparticles with Biological Effectors in An Electrospun Fiber-based Nerve Guidance Conduit to Promote Peripheral Nerve Repair

Binghui Jin[§], Yiling Yu[§], Chenghao Lou, Xiaodi Zhang, Bowen Gong, Jinghao Chen, Xiangxiang Chen, Zihan Zhou, Liqun Zhang, Jian Xiao, Jiajia Xue**

[§]These authors contributed equally to this work.

*Corresponding authors. Email: xfxj2000@126.com; jiajiaxue@mail.buct.edu.cn

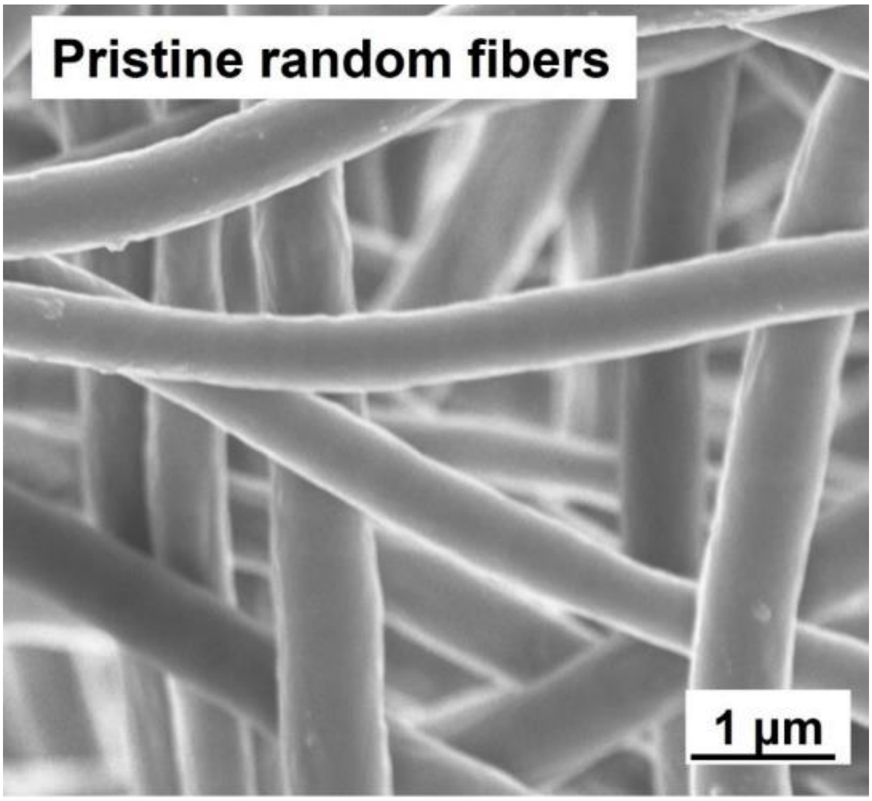

Figure S1. SEM image of pristine random fibers.

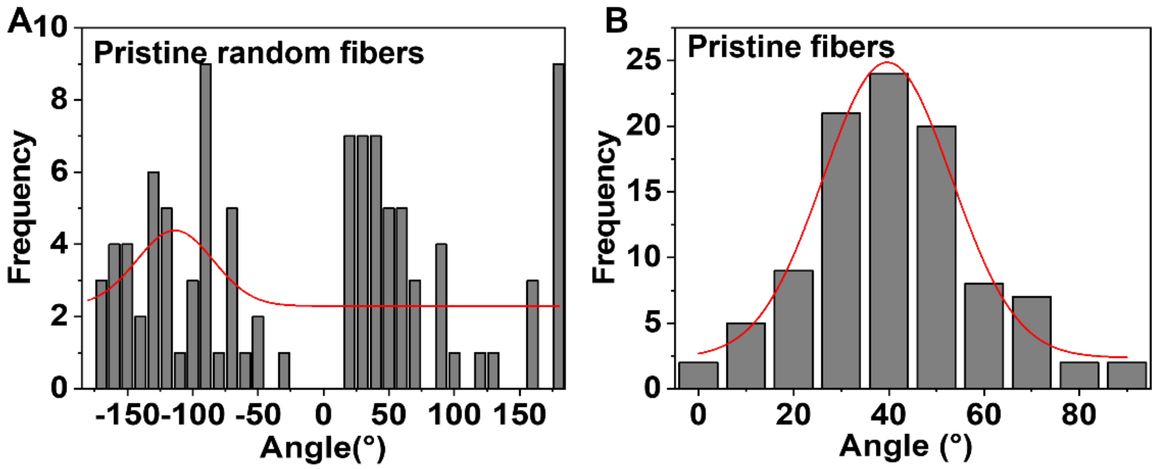

Figure S2. Distribution frequencies of (A) pristine random nanofibers and (B) pristine aligned nanofibers, respectively.

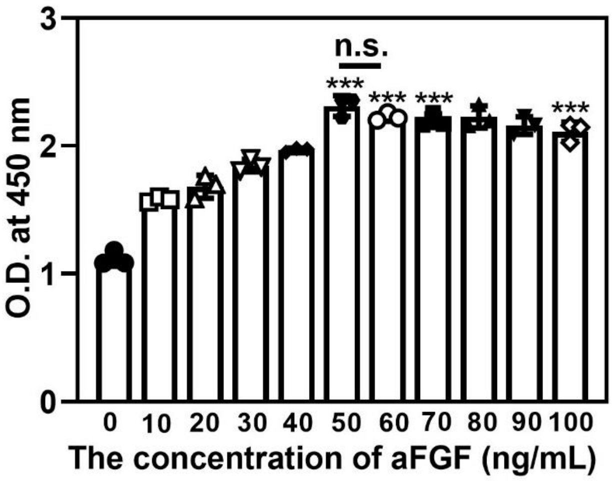

Figure S3. The O.D. of RSC 96 cells after incubation for 5 days with aFGF at different concentrations of 0, 10, 20, 30, 40, 50, 60, 70, 80, 90 and 100 ng/mL ($^{***}p < 0.001$).

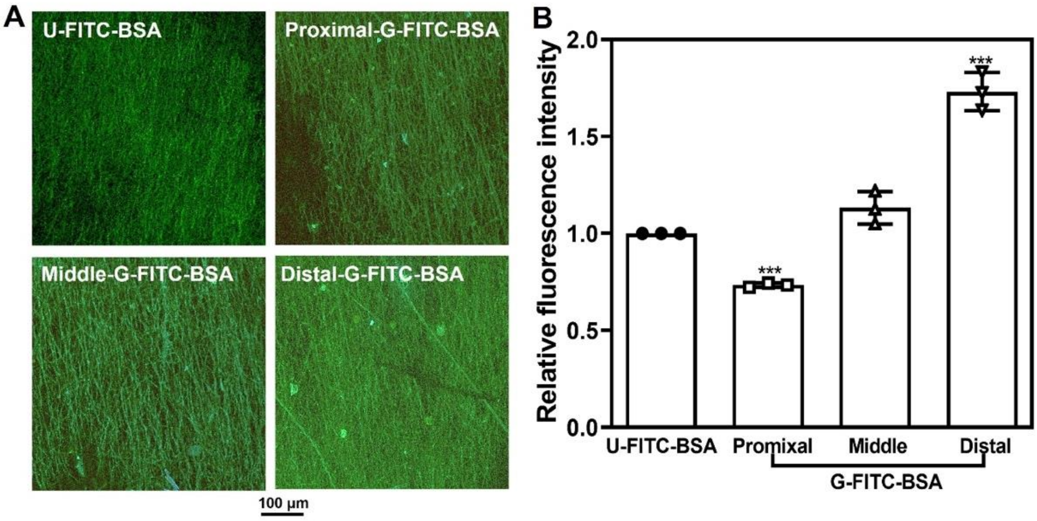

Figure S4. (A) Fluorescence micrographs showing the deposition of FITC-BSA-encapsulated collagen particles on the different types of scaffolds. (B) The relative fluorescence intensities of the different types of scaffolds (n = 3). $^{***}p < 0.001$ in comparison to the group of U-FITC-BSA.

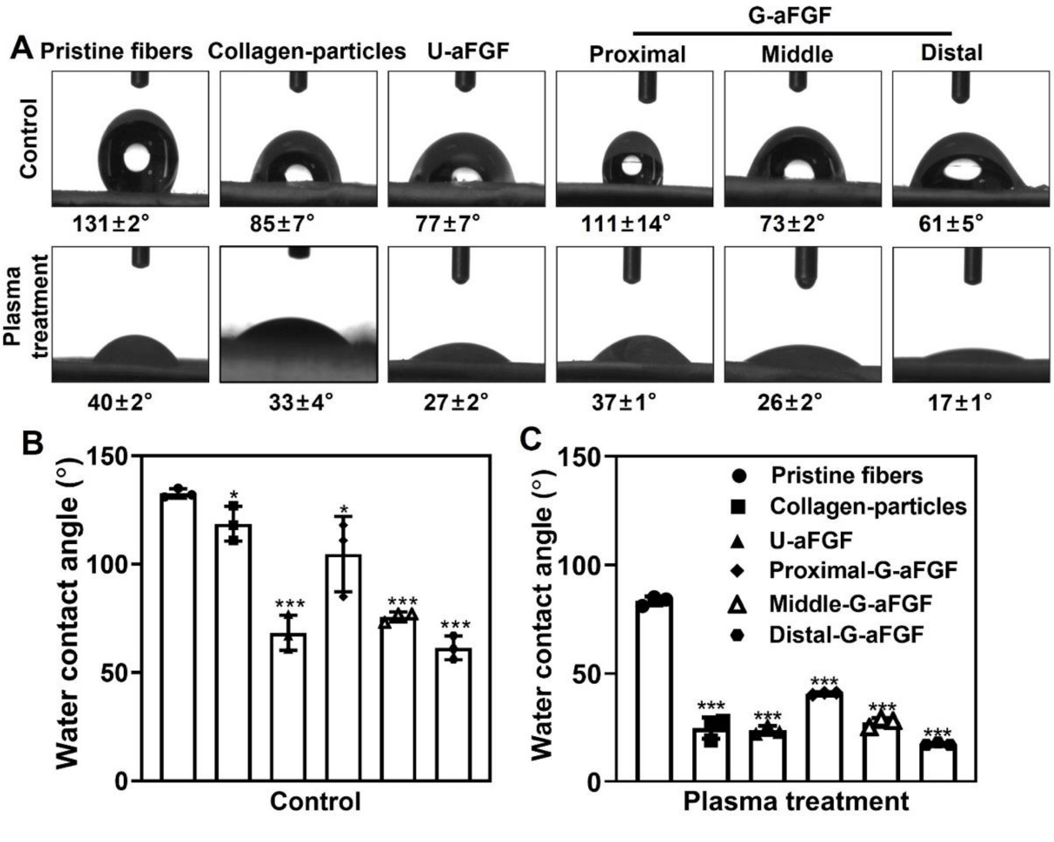

Figure S5. (A)The water contact angles of control and plasma treatment on the different scaffolds. (B) The water contact angles of control on the different scaffolds (n = 3). $^{*}p < 0.05$ and $^{***}p < 0.001$ in comparison to the group of Pristine fibers. (C) The water contact angles of plasma treatment on the different scaffolds (n = 3). $^{***}p < 0.001$.

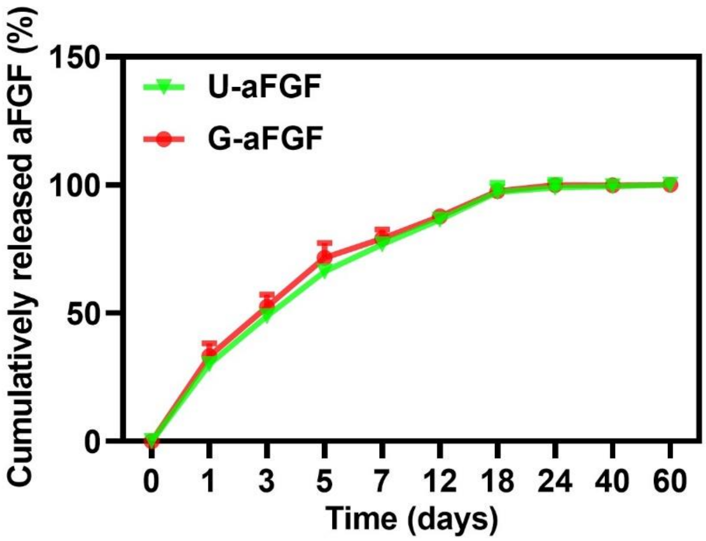

Figure S6. Cumulatively released percentage of aFGF from the U-aFGF and G-aFGF scaffolds after 0, 1, 3, 5, 7, 12, 18, 24, 40, and 60 days.

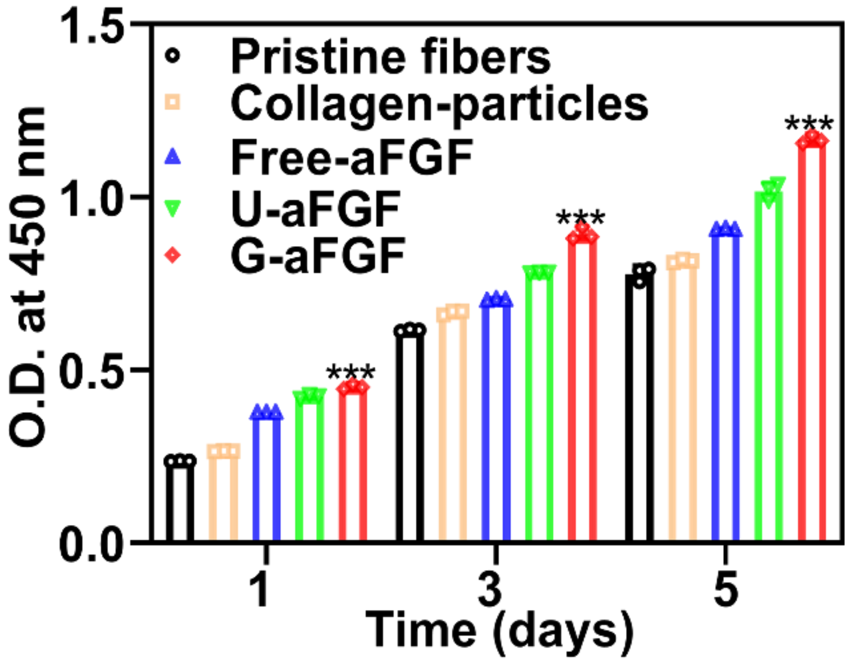

Figure S7. The O.D. of RSC96 cells after proliferation for 1, 3, and 5 days on the different types of scaffolds (n = 3). $^{***}p < 0.001$.

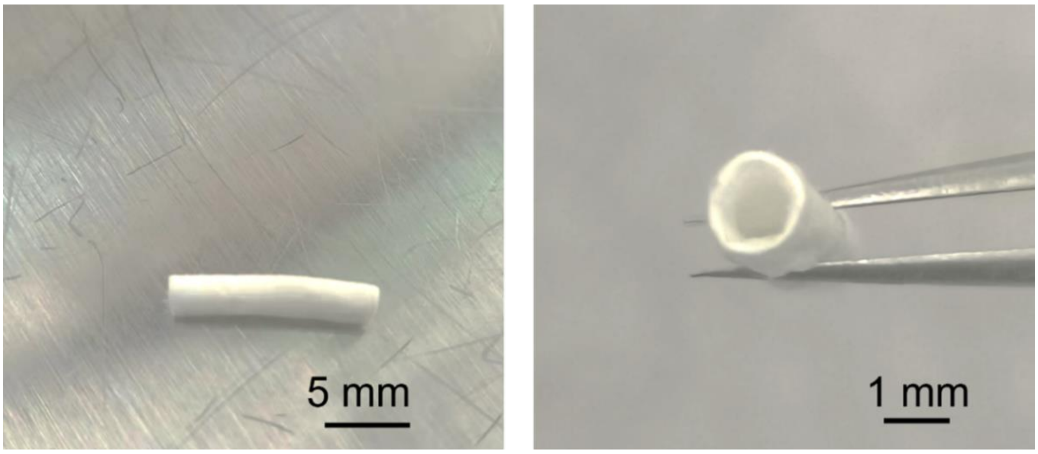

Figure S8. The photo of a typical type of fiber-based NGC.

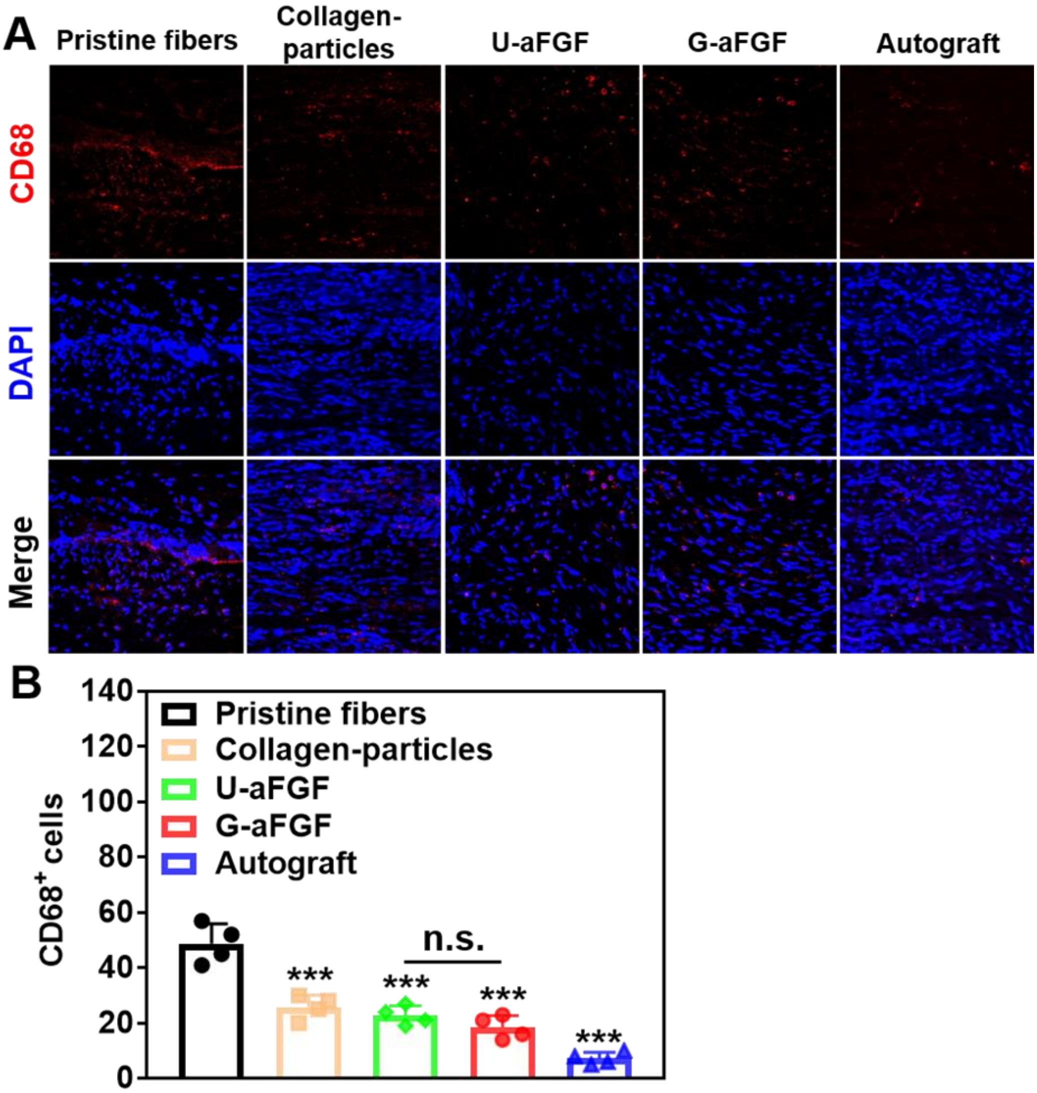

Figure S9. (A) Immunofluorescent images of CD68 of the regenerated nerve in 5 groups at 12 weeks. (B) Statistical analysis of the CD68 positive cells in 5 groups. ***$p < 0.0001$ (n = 4) in comparison to the group of Pristine fibers.
